# Investigating the rise of Omicron variant through genomic surveillance of SARS-CoV-2 infections in a highly vaccinated university population

**DOI:** 10.1099/mgen.0.001194

**Published:** 2024-02-09

**Authors:** Ilinca I. Ciubotariu, Rebecca P. Wilkes, Jobin J. Kattoor, Erin N. Christian, Giovanna Carpi, Andrew Kitchen

**Affiliations:** ^1^​ Department of Biological Sciences, Purdue University, West Lafayette, Indiana 47907, USA; ^2^​ Department of Comparative Pathobiology, Animal Disease Diagnostic Laboratory, Purdue University College of Veterinary Medicine, West Lafayette, Indiana 47907, USA; ^3^​ Purdue Institute of Inflammation, Immunology and Infectious Disease, West Lafayette, Indiana 47907, USA; ^4^​ Department of Anthropology, University of Iowa, Iowa City, Iowa, USA

**Keywords:** genomic surveillance, Omicron, phylodynamics, SARS-CoV-2, sequencing, university population

## Abstract

Novel variants of severe acute respiratory syndrome coronavirus 2 (SARS-CoV-2) continue to emerge as the coronavirus disease 2019 (COVID-19) pandemic extends into its fourth year. Understanding SARS-CoV-2 circulation in university populations is vital for effective interventions in higher education settings and will inform public health policy during pandemics. In this study, we generated 793 whole-genome sequences collected over an entire academic year in a university population in Indiana, USA. We clearly captured the rapidity with which Delta variant was wholly replaced by Omicron variant across the West Lafayette campus over the length of two academic semesters in a community with high vaccination rates. This mirrored the emergence of Omicron throughout the state of Indiana and the USA. Further, phylogenetic analyses demonstrated that there was a more diverse set of potential geographic origins for Omicron viruses introduction into campus when compared to Delta. Lastly, statistics indicated that there was a more significant role for international and out-of-state migration in the establishment of Omicron variants at Purdue. This surveillance workflow, coupled with viral genomic sequencing and phylogeographic analyses, provided critical insights into SARS-CoV-2 transmission dynamics and variant arrival.

## Data Summary

All genomic data generated through this research and used in this study were made publicly available (see Table S1 for individual GISAID accession numbers and full dataset). We also gratefully acknowledge the authors and other submitting laboratories that generated and shared their SARS-CoV-2 viral genomes via GISAID, some of which are used in our analyses (Table S2). Further, our scripts for phylogenomic analyses are made available on GitHub (https://github.com/drupiter/SARS-CoV-2_Purdue).

**Impact Statement**

Severe acute respiratory syndrome coronavirus 2 (SARS-CoV-2) is the etiological agent of COVID-19, which has claimed nearly seven million lives worldwide since its emergence in late 2019. While university students are generally at lower risk of severe disease from SARS-CoV-2, university settings should be investigated further due to their potential impact on neighbouring communities. To fill this research gap and thus expand on the limited research available on SARS-CoV-2 genomic surveillance in university contexts, we utilized whole-genome sequencing to investigate circulating variants at Purdue University from August 2021 to late April 2022. Our study describes the rapid transition from Delta to Omicron variants and uses Bayesian phylogeographic analyses to provide a deeper understanding into introduction events in the university community. This research furthers our understanding and highlights the utility of coupling genome surveillance and bioinformatics tools to study SARS-CoV-2 variant transmission.

## Introduction

The Coronavirus disease 2019 (COVID-19) pandemic caused by the severe acute respiratory syndrome coronavirus-2 (SARS-CoV-2) resulted in severe morbidity and mortality worldwide. As of mid-April 2023, there were over 750 million cases and over six million deaths across the globe [1], with approximately 100 million cases and over one million deaths recorded in the USA alone [2]. SARS-CoV-2 continues to replicate and spread through the world population, and like other viruses, accumulates mutations allowing for new variants to emerge. While most of these mutations do not exhibit concerning characteristics for public health and have little impact on disease severity, some variants have led to enhanced viral fitness [3] as the virus population adapts to the changing immune profile of its human host populations. Potential negative consequences of emerging variants include increased transmission, evasion from diagnostic tests, and reduced efficacy of natural or induced-vaccine immunity [4]. As these factors have important implications for virus transmission and disease control, it is essential to conduct surveillance and monitor circulating variants [4].

The Delta variant (B.1.617.2 and AY lineages) has mutations in the gene encoding the SARS-CoV-2 spike protein causing substitutions T478K, P681R, and L452R, which have led to increased transmissibility. This variant, first identified in India in late 2020, was later classified as a Variant of Concern (VOC) in May 2021 due to its higher transmissibility, susceptibility to monoclonal antibody treatments, and reduced neutralization by post-vaccination sera relative to variants circulating at the time [5, 6]. Moreover, this variant was documented in breakthrough infections of individuals who were fully vaccinated, although those cases were less severe [7]. Nonetheless, this pattern demonstrated that people remain at risk for SARS-CoV-2 even after vaccination (regardless of vaccine type), and further studies are needed to understand transmission in these populations [8]. Ultimately, Delta variant was downgraded to a Variant Being Monitored (VBM) as of mid-April 2022, after surveillance of this variant identified drastically reduced case numbers over the prior months [9].

Delta was reclassified as a VBM because it was rapidly replaced by a variant first identified in November 2021 in South Africa and coined Omicron (B.1.1.529 and BA lineages). The Omicron variant was soon after classified as a VOC by the World Health Organization (WHO) due to epidemiological data showing increased transmissibility and molecular work indicating over 30 spike protein substitutions compared to the original strain [10, 11]. Some of the amino acid substitutions in the spike protein were identified in previous VOCs and are associated with reduction in neutralization by post-vaccination sera and decreased susceptibility to existing monoclonal antibody treatments [12, 13]. Indeed, compared to Delta variant infections, studies on Omicron have shown decreased vaccine efficacy and partial evasion of vaccine-induced immunity, resulting in widely documented breakthrough infections, including individuals with a booster vaccine [14–16]. The Omicron VOC became the dominant variant globally as of late February 2022, accounting for a majority of reported sequenced cases [13]. Notably, the success and rapid expansion of Omicron has led to the establishment of several sublineages, such as BA.1, BA.1.1, BA.2, which have been studied to understand differences in transmissibility among them [17].

Genomic surveillance of SARS-CoV-2 provides insights into virus evolution, transmission dynamics, and the introduction of variants in communities, which facilitates implementation of strategies to mitigate and contain outbreaks [18]. Institutions of higher education are particularly susceptible to rapid viral spread given the size and multitude of congregate settings and social gatherings with high density of people. Furthermore, given the importance of higher education in society and the need to maintain confidence in the safety of students, studying the dynamics of infectious disease in these institutions has been of special interest [19–27]. There have been multiple previous works which have investigated various aspects of SARS-CoV-2 dynamics in university settings since the pandemic began, ranging from genomic epidemiology to modelling studies and routine surveillance [20–24, 26, 28–35]; however, very few studies have explored the transition from Delta to Omicron variants [36–39], let alone in university populations [40].

We believe it is crucial to understand transmission during this time period, as campuses across the United States resumed in-person learning and activities following the implementation of vaccination. Here, we sought to fill this gap by utilizing genomic surveillance to retrospectively characterize SARS-CoV-2 transmission dynamics in a large public university. At the time of this study, approximately 85 % of the Purdue University campus population and 95 % of faculty were vaccinated against SARS-CoV-2 [41], and we aimed to assess the transmission of variants over the course of two semesters in this highly vaccinated community. Using Whole Genome Sequence (WGS) data generated at Purdue University from samples collected between late August 2021 to early April 2022, augmented with publicly available sequence data from the USA and other countries, we describe the rapid transition from Delta to Omicron variants and use phylodynamic analyses to investigate introduction events in this university community.

## Methods

### Campus testing and study population

Purdue University is a large public university located in West Lafayette, Indiana. At the start of the Fall 2021 semester, the Protect Purdue Plan provided guidance for returning to normal operations on campus, which consisted of the option to get fully vaccinated against SARS-CoV-2 with one of the approved vaccines or participate in routine surveillance testing as previously described [19, 42]. Unvaccinated individuals were subjected to testing as frequently as weekly, and targeted testing was conducted as needed to detect any potential hotspots; members of the campus population presenting themselves to campus health centres when feeling sick or after exposure to a positive case were also tested and these test results were also included in the campus surveillance. For each test, metadata were recorded from individual reporting at time of sample collection, including age, gender, symptom status, vaccination status (information such as maker, dose number, and date of most recently received vaccine, as applicable), recent travel history (previous 2 weeks), and whether the individual experienced a previous COVID-19 infection prior to current testing.

The campus testing at the Protect Purdue Health Centre (PPHC) was done by means of rapid antigen tests. The PPHC collected a second anterior nasal swab to be tested for positivity confirmation and sequencing purposes in the Animal Disease Diagnostic Lab (ADDL) at Purdue University, which had certification from Clinical Laboratory Improvement Amendments (CLIA) to perform high complexity testing. The swabs with plastic shafts recommended for PCR testing were collected in PrimeStore molecular transport media (MTM) (Longhorn Vaccines and Diagnostics, Bethesda, MD), which allows for the safe collection, transport, and processing of pathogenic samples as it preserves and stabilizes microbial nucleic acid [43].

### RNA extraction and RT-PCR

Nucleic acids were extracted with the MagMAX Viral/Pathogen Nucleic Acid Isolation Kit (Applied Biosystems, Thermo Fisher Scientific, Waltham, MA, USA) with a KingFisher Flex Purification System (Thermo Fisher Scientific, Waltham, MA, USA). Nucleic acid RT-PCR was performed using the Thermo Fisher TaqPath COVID-19 Combo Kit (Applied Biosystems, Thermo Fisher Scientific, Waltham, MA, USA) on a 7500 Fast Real-Time PCR System (Applied Biosystems, Thermo Fisher Scientific, Waltham, MA, USA). This multiplex kit targets three regions of SARS-CoV-2 virus, namely the ORF1ab, N, and S genes – the latter of which has been used as an indicator of variants with a 69-70del S-gene mutation, such as Alpha and Omicron [44, 45].

### Whole-genome sequencing using Ion Torrent system

#### Selection of samples for sequencing

We sequenced 793 SARS-CoV-2 positive samples collected in Fall 2021 and Spring 2022, to represent two complete university semesters on Purdue’s campus. Following guidance provided by the PPHC regarding which samples to prioritize for sequencing, we specifically targeted individuals who had been vaccinated, individuals with travel history, and a few samples from individuals enrolled in screening who had not been vaccinated. For the purposes of this study, we defined vaccinated individuals as those who received a full series of approved vaccines (i.e. Pfizer, Moderna, J and J, etc.), patients who received an incomplete series (one dose of either Pfizer or Moderna), and those who received booster shots (either Pfizer or Moderna). Additionally, we focused on individuals who had provided recent travel history (domestic or international, within last 2 weeks prior illness) in order to place phylodynamic analyses in context. Following RT-PCR, we conducted viral genomic sequencing of SARS-CoV-2 positive samples with a high quantity of generated RNA and a threshold of ORF1ab CT <30.

#### RT for Ion Torrent sequencing

The extracted samples were reverse transcribed using Ion Torrent NGS reverse transcription kit (Thermo Fisher Scientific, Waltham, MA, USA). The reaction was set up in a 96-well, eight-barcode Ioncode plate (Thermo Fisher Scientific, Waltham, MA, USA). Briefly, 7 µl of sample was mixed with 2 µl of Ion Torrent NGS 5X Reaction Buffer and 1 µl of Ion Torrent NGS 10X RT Enzyme Mix; the mixture was incubated in a thermocycler for 10 min each at 25 and 50 °C before inactivating the reverse transcriptase by incubating it for 5 min at 85 °C. The volume of each sample was increased to 15 µl using nuclease-free water before proceeding to the library prep step.

#### Library prep and Ion 530 chip loading

Library prep for each set of eight samples was automated in an Ion Chef using an Ion AmpliSeq Kit for Chef DL8 (Thermo Fisher Scientific, Waltham, MA, USA). The reagents for library prep were prepared according to the manufacturer’s protocol (Thermo Fisher Scientific, Waltham, MA, USA). In short, 150 µl of 2X Ion Ampliseq SARS CoV-2 Insight Research Assay-GS Chef-ready primer pool one and primer pool two were added into the tubes at position A and position B of the reagent cartridge. All other consumables were loaded on to the Ion Chef according to the manufacturer’s protocol. A specific plan for executing library prep in the Ion Chef was prepared in the Torrent Suite Software (TSS) v 5.12.1 according to the protocol. Amplification conditions in the Ion Chef were set as 4 min for annealing and extension, and the cycle number was set to 19 cycles. Each automated library prep produced 700 µl of 100pM libraries for eight samples. For loading an Ion 530 chip, 12.5 or 25 µl of each of four or two library preps were used, respectively. Chip loading was performed with an Ion 510 and Ion 520 and Ion 530 Kit (Thermo Fisher Scientific, Waltham, MA, USA) according to the manufacturer’s protocol. In the plan for chip loading, prepared in the TSS, plugins for analysis explicitly developed for SARS-CoV-2 by Thermo Fisher Scientific were used (pipeline: generateConsensus, SARS_CoV_2_lineageID, SARS_CoV_2_CoverageAnalysis, SARS_CoV_2_annotateSnpEff, and SARS_CoV_2_VariantCaller). BED files for Ion Ampliseq SARS-CoV-2 Insight reference library and target regions were also added into the plan. Prepared library preps were loaded into positions one and two of the reagent cartridge, and the rest of the reagents were loaded onto the Ion Chef; chip loading was performed according to the manufacturer’s protocol.

#### Sequencing and lineage assignment

Sequencing was performed on an Ion S5 semiconductor sequencer (Thermo Fisher Scientific, Waltham, MA, USA) using the Ion 510 and Ion 520 and Ion 530 kits according to the manufacturer’s protocol. Aligned files were obtained as BAM format from the TSS; these files were converted to FASTQ using the FileExporter plugin of TSS, and then FASTA files were used for downstream analysis. Phylogenetic lineages were assigned to genomic sequences by the SARS-CoV-2 Insight panel workflow and were double-checked using the PANGOLIN lineage tool v.4.0.1/PLEARN-v1.2.133 [46].

### Data curation

FASTA consensus sequences were evaluated for unexpected frameshifts (due to indel sequencing errors) using Nextclade v.2.1.0 (https://clades.nextstrain.org/) and errors were manually corrected. Corrected FASTA files of the consensus sequences were deposited to the Global Initiative on Sharing All Influenza Data (GISAID), aimed at rapid sharing of and open access to epidemic and pandemic virus data [47, 48]. These sequences are accessible by searching ‘hCoV-19/USA/IN-LH’ from 23 August 2021 to 2 May 2023.

### Selection of publicly available genomes for analyses

To compare patterns of lineages in the same time frame from locations such as the state of Indiana and the United States, we downloaded and included publicly available sequences from GISAID. Specifically, our search on GISAID was performed using the ‘Location’ identifier for Indiana state and the United States in the North American continent and simultaneously the ‘Collection date’ to include the same 32 week period starting on 22 August 2021 and ending on 2 April 2022. We selected the sequences from the state of Indiana (Virus names hCoV-19/USA/IN-) and the United States (Virus names hCoV-19/USA-) for further analyses.

### Phylogeographic and clustering analyses

Phylogenetic analysis of genome sequences derived from positive cases sampled amongst the Purdue community was performed to characterize the origin of viruses circulating in the Purdue population. Genome sequences were aligned using the Nextalign CLI tool [49] with the Wuhan-Hu-1 sequence [50] (Genbank accession NC_045512) as a reference guide. A preliminary analysis of all samples was performed using the BEAST v1.10.4 package [51, 52], with a strict molecular clock (rate=7.5×10^−4^ substitutions per site per year [53], a constant population size demographic model, and a Hasegawa, Kishino, Yano (HKY) substitution model [54] with a gamma distribution to account for site-rate heterogeneity [55]. Markov chains were run for at least 250 million generations, sampled every 10 000 generations, and in duplicate to ensure sufficient sampling and convergence. The resulting combined output files from the BEAST analyses were analysed to assess convergence, and the distribution of trees was summarized with the TreeAnnotator function in BEAST using the maximum clade credibility (MCC) criterion to identify the topology, the distribution mean to characterize node heights, and the first 10 % of the Markov chain samples discarded as burn-in.

Due to the overwhelming abundance of SARS-CoV-2 sequences available in publicly accessible databases, phylogeographic analysis of the Purdue genomes was performed on phylogenetically informed subsamples of Purdue genomes. Subsamples were clusters of samples identified from the MCC tree produced by the preliminary analysis of the Purdue genomes. For each cluster of Purdue genomes, closely related genomes were identified by AudacityInstant (v5.0.1; www.epicov.org) and downloaded from GISAID [56], and then combined with the Purdue genomes to produce an alignment capable of properly contextualizing the Purdue cluster for phylogenetic analysis. Each combined sample of genomes was aligned as above (i.e. using Nextalign), and curated for completeness and uniqueness with custom PERL scripts. The combined datasets of Omicron sequences identified by AudacityInstant (GISAID) were sufficiently large to make phylogenetic inference intractable, so duplicate virus samples that were identical in sequence, sampling date, geographic region, and names were pruned from datasets prior to phylogenetic analysis. Profiles of analyses and viruses from each cluster are available as Supplemental Materials (see Tables S3 & S4).

Phylogeographic analysis of each cluster of genome sequences (Purdue genomes and contextual sequences from GISAID) were analysed in BEAST v1.10.4 to produce posterior distributions of trees. Each BEAST analysis was run similarly to the analysis of just Purdue genomes: HKY substitution model with site-rate variation (either a gamma distribution or a gamma distribution plus proportion of invariant sites), a constant population size, a strict clock (rate=7.5×10^–4^ substitutions per site per year), and Markov chains run for at least 250 million generations and duplicated to ensure convergence. Log files for all analyses were assessed for convergence in Tracer v1.7.2 [57], with multiple converged runs combined after the elimination of burn-in using LogCombiner (v1.10.4). The resulting posterior distributions of trees for each cluster of Purdue genomes (with contextual genomes) were then analysed in BaTS (Bayesian Tip association Significance) package [58] to infer the geographical origins of genomes circulating in the Purdue community.

Parsimony scores (PS) represent the number of character changes upon a tree, and thus may be used to identify the number of transitions, migrations, or connections between samples from geographic states. To identify connections between geographic regions in viral phylogenies, PS values were calculated using three- and two-state coding: Purdue, location X, and not-Purdue for three-state coding and combined Purdue and location X versus not-Purdue for two-state coding. The difference in parsimony score between the coding schemes is a measure of how frequently viruses from Purdue and location X cluster, and therefore serves as an estimate of how many introductions may have occurred from location X to (or from) Purdue present in the alignment. PS values were calculated in this manner for each non-Purdue location represented in the dataset. This approach was previously described in an analysis of circulating Gamma variant viruses [19].

All tree visualisations were performed in FigTree, version 1.4.4 (https://github.com/rambaut/figtree/releases).

## Results

### SARS-CoV-2 molecular testing and sequenced cases

During the Fall semester of 2021 from early August to the end of December, there were 91 673 total SARS-CoV-2 tests performed at the Protect Purdue Health Centre. Of these tests, there were 1570 (1.7 %) positive cases. In the Spring semester of 2022, from early January to early April, there were 33 673 tests performed, yielding 4095 (12.2 %) positive cases. A total of 5665 samples (4.5 %) were characterized as SARS-CoV-2 positive during the entire study period, and from these, we selected and generated whole genome sequences for 793 (14 %) total SARS-CoV-2-positive samples (446 collected during the Fall 2021 semester, and 347 during the Spring 2022 semester). Genomic sequencing of these 793 individuals revealed 337 Delta cases and 455 Omicron cases (Table 1). There was only one other variant identified in this collection (Mu) for a male individual who was in the 18–30 age group, vaccinated, symptomatic, with no previous infection; this individual was not included in further analyses specifically aimed at looking more closely into Delta and Omicron cases.

**Table 1.** Characteristics of patients with confirmed SARS-CoV-2 Delta and Omicron variants in Purdue University campus community from sequenced genomes collected 22 August 2021 to 2 April 2022

	Delta* (*N*=337)	Omicron† (*N*=455)
**Collection date range**	23 August 2021 to 21 December 2021	14 December 2021 to 2 April 2022
Age group, N (%)		
<18	5 (1.5)	8 (1.8)
18–30	209 (62.0)	357 (78.5)
31–44	64 (19.0)	48 (10.5)
45–59	47 (13.9)	32 (7.0)
60+	12 (3.6)	10 (2.2)
Sex, N (%)		
Female	169 (50.1)	227 (49.9)
Male	166 (49.3)	218 (47.9)
Unknown	2 (0.6)	10 (2.2)
Patient Symptom Status, N (%)		
Asymptomatic	64 (19.0)	85 (18.7)
Symptomatic	273 (81.0)	370 (81.3)
Vaccination Status, N (%)		
Vaccinated‡	317 (94.1)	404 (88.8)
Primary series complete	297 (88.1)	221 (54.7)
*BNT162b2*: BioNTech/Pfizer	214 (72.1)	148 (67.0)
*mRNA-1273*: Moderna	54 (18.2)	43 (19.5)
*Ad26.COV2.S*: Johnson and Johnson	21 (7.1)	13 (5.9)
*AZD1222*: AstraZeneca/Oxford	1 (0.3)	1 (0.5)
*ChAdOx1*: Serum Institute of India	3 (1.0)	9 (4.1)
*BBIBP-CorV*: Sinopharm	2 (0.7)	3 (1.4)
*CoronaVac*: Sinovac Biotech	2 (0.7)	1 (0.5)
*BBV152*: Bharat Biotech	0 (0)	3 (1.4)
Days since primary series, Mean (SD)	182.6 (53.9)	216.7 (68.3)
Partially complete primary series: Pfizer	3 (0.9)	3 (0.7)
Booster Dose	17 (5.4)	180 (44.6)
*BNT162b2*: BioNTech/Pfizer	14 (82.4)	157 (87.2)
*mRNA-1273*: Moderna	3 (17.6)	23 (12.8)
Days since booster shot, Mean (SD)	16.2 (25.5)	61.3 (35.0)
Not vaccinated	17 (5.0)	45 (9.9)
Not stated	3 (0.9)	6 (1.3)
Previous Infection§, N (%)		
>90 days earlier	8 (2.4)	57 (12.5)
<90 days earlier	11 (3.3)	11 (2.4)
No	317 (94.1)	381 (83.7)
Not stated	1 (0.3)	6 (1.3)
Previous travel history, N (%)		
Yes	79 (22.4)	148 (34.2)
No	273 (77.6)	285 (65.8)

*Includes original Delta variant B.1.617.2 and all AY lineages.

†Includes all Omicron BA lineages observed.

‡Includes all individuals with one, two, or three doses of Pfizer, Moderna, J and J, etc. vaccines.

§Previous SARS-CoV-2 infection confirmed by previous positive test.

### Demographic and clinical characteristics

Individuals with confirmed Delta variant infections had a median age of 22 years (range 4–72) and the median age of individuals with Omicron variant infections was also 22 (range 3–78) (Table 1). Approximately the same ratio of asymptomatic and symptomatic patients at the time of sample collection was observed for both Delta and Omicron infections (Chi-square; *P*>0.05): 19.0 % asymptomatic and 81.0 % symptomatic for Delta, and 18.7 % asymptomatic and 81.3 % symptomatic for Omicron (Table 1). No individuals had documented prior infections both more than 90 days and less than 90 days prior to current infection.

SARS-CoV-2 vaccination status was recorded at the time of swabbing for 334 (99.1 %) of Delta cases and 449 (98.7 %) of Omicron cases. Of these with known vaccination status, there were 297 (88.1 %) individuals infected with Delta and 221 (54.7 %) individuals infected with Omicron that had received a completed primary series of a specified vaccine (Table 1). Additionally, 17 (5.4 %) individuals identified with Delta variant and 180 (44.6 %) with Omicron had received a booster of either the Pfizer or Moderna vaccines. Seventeen (5.0 %) individuals infected with Delta variant and 45 (9.9 %) with Omicron were unvaccinated. Most individuals, regardless of variant infection, completed their primary vaccine series by the start of the study period (only six individuals or 0.8 % had partial series administered). Moreover, the number of days since the administration of primary series for Omicron cases (mean 216.7) was higher than for Delta cases (mean 182.6) (Mann-Whitney-Wilcoxon; *P*<0.05), and the same pattern was observed for boosted individuals (average 61.3 days for Omicron, 16.2 days for Delta) (Mann-Whitney-Wilcoxon; *P*<0.05).

### Transition from Delta to Omicron variants

**Fig. 1.**
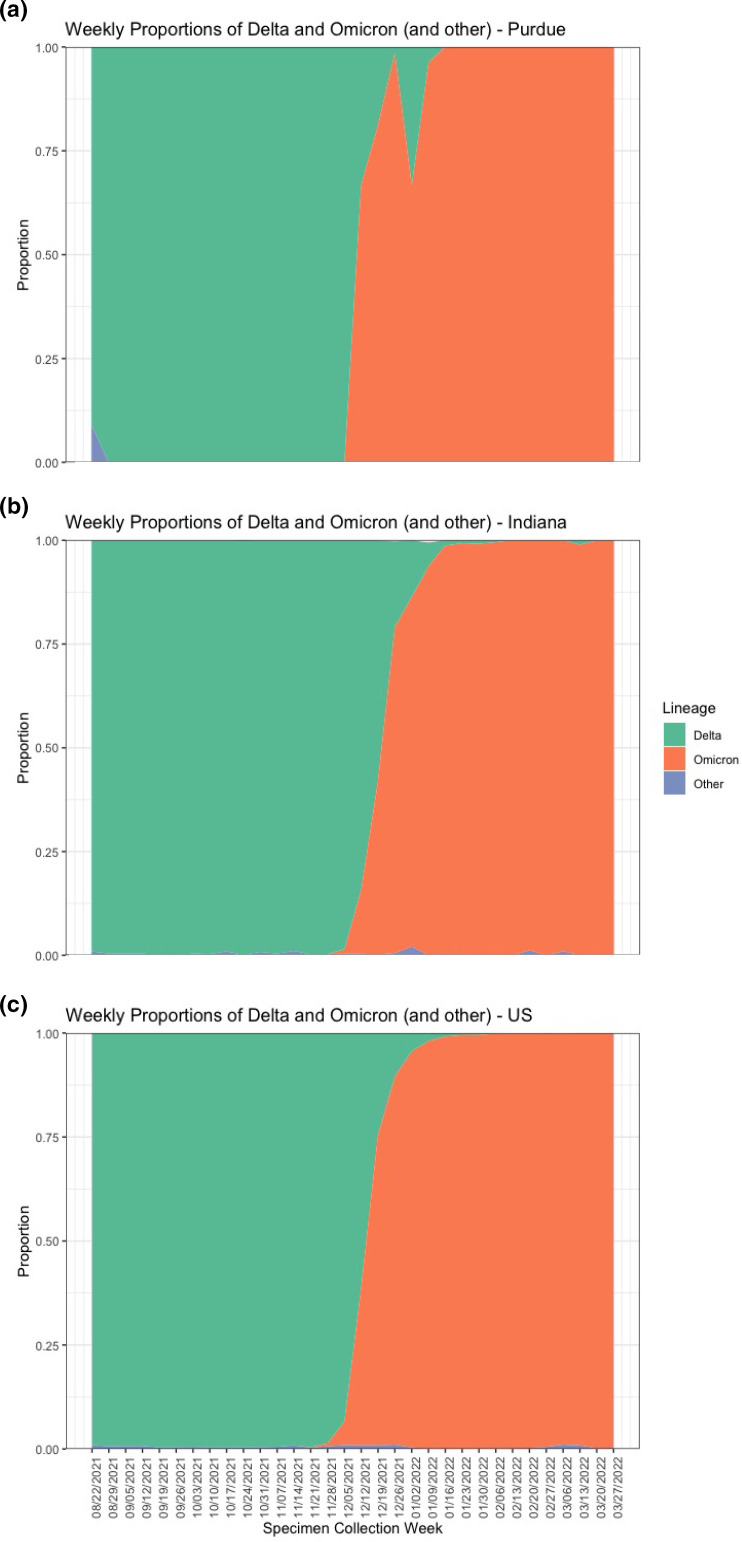
Proportion of Delta, Omicron, and other lineages identified in sequenced cases per week during a 32 week period (22 August 2021 to 2 April 2022) as of 15 November 2022 on GISAID. (**a**) Proportion of cases sequenced per week at Purdue University that were identified as Delta variant (B.1.617.2 and AY lineages), Omicron (BA.1, BA.2, and sublineages), and other variants (such as Mu, Gamma, etc.). (**b**) Proportion of weekly sequenced cases identified as Delta, Omicron, or other from the rest of Indiana state as acquired from publicly available data on GISAID. (**c**) Proportion of weekly sequenced cases identified as Delta, Omicron, or other from the rest of the USA as acquired from publicly available data on GISAID.

In all three geographic contexts (campus community, state of Indiana, and the United States as a whole) which we examined during the same time frame of the study, Delta dominated the variant landscape in the early weeks of the study, with very few other lineages detected from sequenced cases (Fig. 1a-c). However, once the first few cases of Omicron were identified, the proportion of Delta infections was quickly observed to decrease (Fig. 1a-c). We detected the first Omicron variant sequenced case on Purdue campus on 14 December 2021, during week 17 of the 32 week study period (Fig. 1a). This was delayed when compared to the time the first Omicron variant case was sequenced and made available on GISAID both in the state of Indiana and the United States, or weeks 16 and 11, respectively (Fig. 1b, c). In the first week when Omicron was detected by sequencing at Purdue University, the proportion of this variant represented 66 % of sequenced cases, with a rapid increase to 100 % prevalence in the following 5 weeks (Fig. 1a). The state of Indiana detected the presence of Omicron 1 week earlier, during which the proportion was less than 10 %, followed by a rapid increase to over 75 % of sequenced cases in the following 3 weeks, and over 90 % by week 21 (Fig. 1b). Similarly, the United States detected the first Omicron case from samples sequenced during week 11, and this proportion when compared to Delta variant or other identified variants remained at low levels until it reached over 50 % during week 18 (Fig. 1c). Of note, week 20 saw a decrease in the proportion of sequenced Omicron cases at Purdue because there were only three collected specimens in this time frame due to low presence on campus community following the New Year holiday (Fig. S1, available in the online version of this article).

### Phylogenetic analysis of Purdue community viruses

**Fig. 2.**
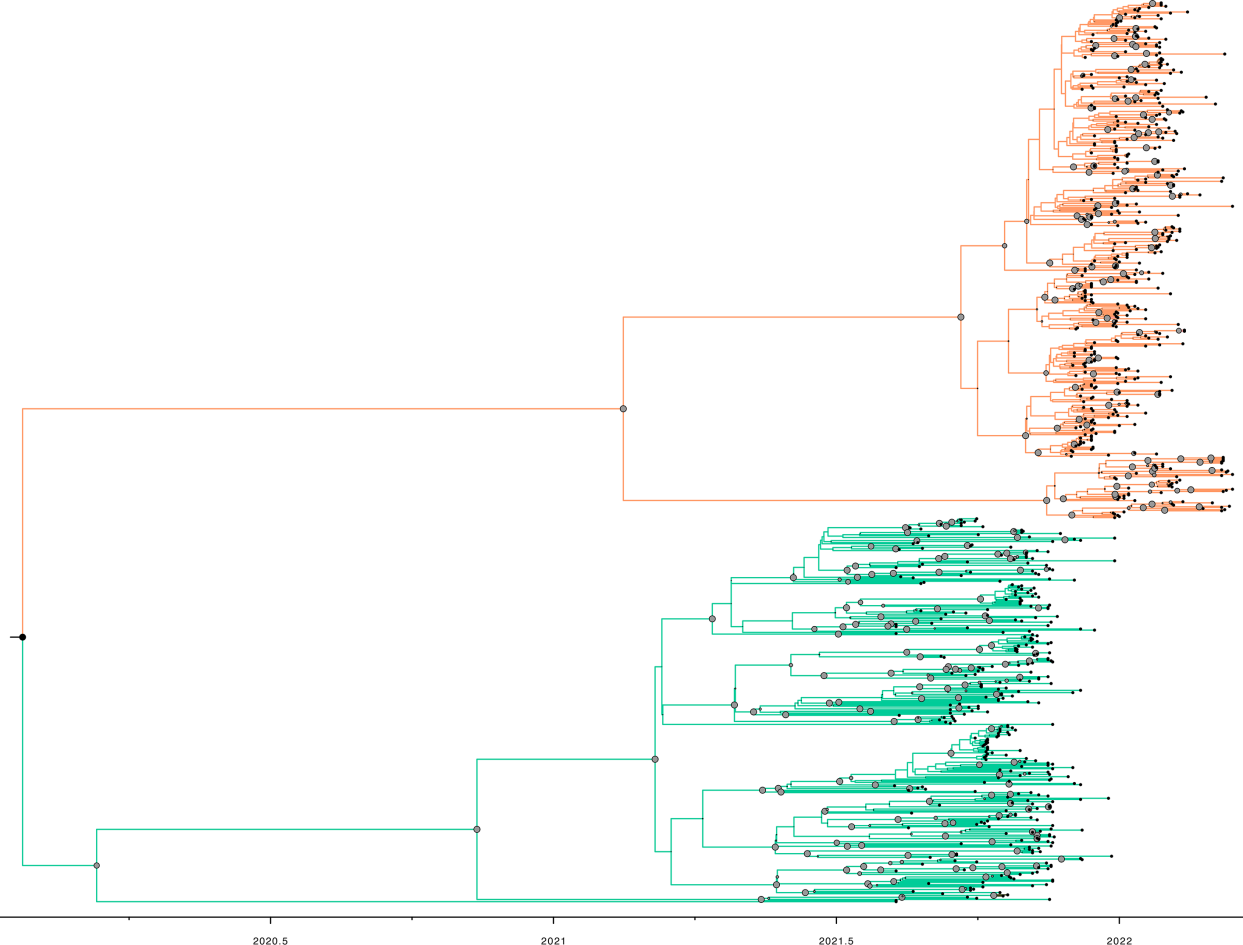
Bayesian phylogeny from 793 SARS-CoV-2 genomes generated from Purdue community. This presents a time-informed Maximum Clade Credibility (MCC) tree of the 793 genomes successfully generated from samples collected at Purdue University during the academic year from early August 2021 until early April 2022. The tree used an HKY +G nucleotide substitution, constant population size, and strict molecular clock model and shows the replacement of Delta variant sequences by Omicron over time. Each sequence in the tree is highlighted by a small black tip. The two main colours correspond with the scheme shown in Fig. 1 with samples shown in green denoting samples identified through sequencing as Delta, while the ones in orange are Omicron confirmed samples. The grey coloured dots in the middle of the branches show high support bootstrap values (>90 %).

We performed a phylogenetic analysis of the 793 SARS-CoV-2 genomes generated from samples taken from the Purdue community. The resulting Bayesian phylogeny (Fig. 2) clearly shows the division of the Purdue sequences into Delta and Omicron clades with strong support (posterior support=1.00), as well as the replacement of Delta variant sequences by Omicron sequences during late 2021. The internal structure of each variant lineage displayed uniformly lower support, which is consistent with both the rapid diffusion of Delta and Omicron variants and the depth of sampling; when combined with a relatively low substitution rate for RNA viruses [59, 60], rapid diffusion and deep sampling may produce large and poorly resolved viral genealogies. Information about the number of Purdue and contextualizing GISAID sequences and demographic, substitution, and clock models for each cluster analysis are available in Tables S3 and S4, whilst the breakdown of Purdue sequences by cluster is presented in Tables S5 and S6.

**Fig. 3.**
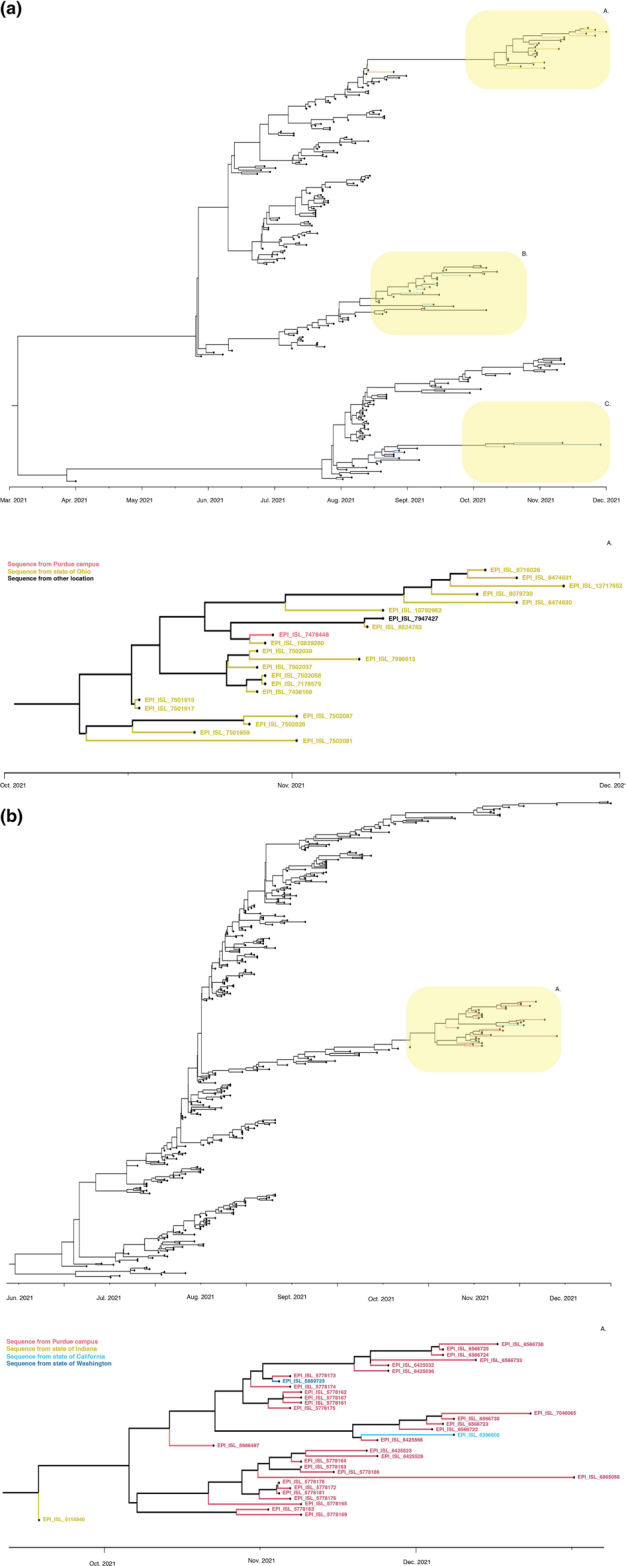
BaTS Delta Clusters: Bayesian maximum clade credibility tree (MCC). This tree was generated in BEAST using HKY+G+I model. Here, we specifically highlight a second type of observed phylogenetic relationship comprised of three clusters and 237 taxa (corresponding group 21 in Table S4). In the bottom half of the panel, we present a zoomed-in view of the top yellow shaded area, showing mostly local transmission from the state of Ohio (a). (b) BaTS Delta Clusters: Bayesian maximum clade credibility tree (MCC). This MCC tree was generated in BEAST using HKY+G+I model. Here, we specifically highlight one type of observed phylogenetic relationship comprised of three clusters and 346 number of taxa (corresponding group 17 in Table S4). In the bottom half of the panel, we present a zoomed-in view of the yellow shaded area with support for introduction events from state of Indiana, Washington, California.

A parsimony score-based method was used to infer the relationships between the populations of SARS-CoV-2 viruses at Purdue and viral populations found elsewhere. To do this, the Purdue genomes were divided into clusters using the Bayesian phylogeny in Fig. 2; each cluster of genomes was contextualized by identifying closely related genomes that were downloaded from GISAID [56]. The PS differences used to infer connections between the Purdue SARS-CoV-2 population and the viral populations from other geographic regions are presented in (Fig. 3) (Delta).

### Clustering results

For the clustering analysis using PS, we tracked PS differences (three-state PS minus two-state PS) ≥ 0.05 for each cluster of Purdue sequences. Because clusters were of different sizes, we summed the PS differences for each region (i.e. Purdue and region ‘X’) across all clusters of Delta or Omicron sequences. The summed PS differences were used as statistical indicators of connections between Purdue and other geographic regions.

The summed PS differences for Delta and Omicron are summarized in Tables 2 and 3 for connections between Purdue and USA states and countries, respectively (with full results displayed in Tables S1 & S2, and S7 & S8). Notably, for Delta variant viruses, only three of these potential connections are supported by summed PS differences≥1.0 (Canada, Slovenia, and the United States), whilst 13 were≥1.0 for Omicron variant viruses (Bangladesh, Brazil, Canada, Germany, India, Morocco, Netherlands, Panama, Poland, Spain, Sweden, United Kingdom, and the United States), with few countries (*n*=2) shared between the Delta and Omicron sets (only Canada and the United States) (Table 2 & S1). We note that the use of the cut-off of PS≥1.0 reflects our previous analysis of Gamma variant viruses that identified strong Bayesian support for PS values>1.0 [19]. We therefore constrain our interpretation of connections with associated PS values≥1.0.

**Table 2.** Summed Parsimony-Score (PS) differences ≥ 1.0. Clustering analysis results using parsimony-score based method for non-USA countries with PS inferred connections to Purdue (see Table S1 for full detailed results of all countries’ scores). Table displays results for both Delta and Omicron variant analyses

Country name	Delta	Omicron
**Bangladesh**	–	2.422
**Brazil**	–	1.285
**Canada**	2.427	3.217
**Germany**	–	4.361
**India**	–	16.077
**Morocco**	–	1.545
**Netherlands**	–	5.732
**Panama**	–	1.532
**Poland**	–	22.089
**Slovenia**	1.000	–
**Spain**	–	2.102
**Sweden**	–	1.139
**United Kingdom**	–	31.501
**USA**	249.228	119.341
***Total***	252.655	212.343

**Table 3.** Summed Parsimony-Score (PS) differences ≥ 1.0. Clustering analysis results using parsimony-score based method for USA states with PS inferred connections to Purdue (see Table S2 for full detailed results of all states’ scores). Table displays results for both Delta and Omicron variant analyses

State name	Delta	Omicron
Alabama	–	2.829
Arizona	1.346	2.730
Arkansas	–	1.100
California	11.513	18.269
Colorado	3.105	8.384
Connecticut	1.723	–
Delaware	–	1.131
Florida	2.74	2.994
Georgia	2.021	1.518
Illinois	25.17	10.074
Indiana	142.228	14.096
Iowa	–	1.402
Kentucky	3.707	–
Massachusetts	4.929	3.089
Michigan	7.987	4.582
Minnesota	2.465	1.610
Montana	1.156	–
New Jersey	–	1.031
New York	2.355	5.856
North Carolina	1.584	2.962
North Dakota	1.181	1.004
Ohio	11.114	–
Oklahoma	–	1.261
Oregon	1.36	5.393
Pennsylvania	2.007	1.233
South Carolina	1.176	–
Tennessee	3.874	9.544
Texas	1.561	5.035
Utah	–	1.180
Virginia	1.039	1.289
Washington	2.203	–
Wisconsin	4.816	2.064
***Total***	244.36	111.66

There was substantially more overlap between the sets of states/territories within the United States with summed PS score differences≥1.0 for Delta (*n*=25) and Omicron variant viruses (*n*=26) (see Table 3 and S2). Specifically, 19 were found in both sets, 13 were found in only one set (*n*=6 for Delta, *n*=7 for Omicron), and 18 USA states were not in either set. For the USA states, comparison of the set of PS difference scores for Delta and Omicron viruses differed statistically (Chi-square; *P*<0.001). Similarly, comparisons of the total domestic (USA states and territories) and foreign (i.e. non-USA) summed PS differences was also significant (Chi-square; *P*<0.05), although not as pronounced, but still indicating more connections between foreign locations and Purdue during the Omicron wave than the Delta wave. Notably, the proportion of all USA-associated PS differences attributable to connections between Purdue and Indiana was 0.571 (142.2 Indiana / 249.2 all USA) for Delta and 0.118 (14.1 Indiana / 119.3 all USA) for Omicron, suggesting Indiana was a much more significant source of viruses migrating into the Purdue community during the wave of Delta viruses than the subsequent early wave of Omicron viruses (although we recognize this could be partly due to Delta variant already being well-established in the studied communities at the time of investigation).

Though it has been shown that sequencing error and selection have produced sites within SARS-CoV-2 alignments that are homoplasic and confound accurate phylogenetic inference (e.g. https://virological.org/t/issues-with-sars-cov-2-sequencing-data/473), we did not exclude these sites from our analysis. Theoretically, the incorporation of site-specific rate heterogeneity in our phylogenetic analysis, the use of Bayesian methods that produce distributions of reasonable/probably trees, and the calculation of parsimony scores integrating across the distribution of trees all combine to both accommodate the uncertainty of including such sites in the analysis and incorporate that uncertainty in our results. To determine if this intuition may be correct, we re-analysed the alignments for two clusters of Delta viruses (clusters 070 and 071) in BEAST and masked a set of previously identified problematic sites (available here https://github.com/W-L/ProblematicSites_SARS-CoV2/blob/master/problematic_sites_sarsCov2.vcf), and calculated PS values for the regions that had PS values≥0.05 in our original analyses. We calculated a strong correlation between the PS values of the unmasked and masked analyses (R=0.998 and R=0.986 for clusters 070 and 071, respectively). Furthermore, as any potential bias is unlikely to be geographically linked across the entire phylogeny, the result of such unaccounted for homoplasy should be random with regard to geographic region, and produce more diffuse PS values than expected due to the isummation of contributions from multiple associations across a single tree to the total PS for a region. In this way, our focus on only the largest PS values is likely to make our interpretation of the overall pattern of parsimony scores conservative.

When examining the full data set for travel history, we found that out of all samples, 227 individuals (28.7 %) reported that they had travelled prior to their SARS-CoV-2 positive test, while 558 people (70.4 %) indicated that they did not travel and eight individuals (1.0 %) did not answer the question (Table 1). Out of the 227 individuals who reported travel history (see Fig. S2 for full map of travel data), 79 (34.8 %) had Delta variant and 148 (65.2 %) had Omicron variant (Table 1). From the individuals with Delta variant, eight (10.1 %) reported out-of-country travel to eight separate countries, while the remaining 71 persons travelled in other states throughout the USA. In contrast, 36 (24.3 %) individuals who tested positive with Omicron variant reported out-of-country travel to 16 different countries. For both types of variant infections, most individuals had reported travel to Illinois (25.3 % for Delta and 22.3 % for Omicron) with the second most prevalent location being Ohio for Delta variant (11.4 %) and Florida for Omicron variant (9.5 %).

## Discussion

As genomic surveillance capacity and sequencing availability increased throughout the Covid-19 pandemic, genomic epidemiology offered the opportunity for surveillance of viral lineages, monitoring spread of variants, and continued research into virus evolution [61–65]. Our SARS-CoV-2 genomic study offers a more comprehensive understanding of viral lineage transmission and introduction events in a university community during a period when university operations returned to normal operation and in the presence of high vaccination rates (~89 %) amongst the university population [66].

Overall, we sequenced 793 total cases throughout the Fall and Spring semesters, providing insight from multiple perspectives, such as demographic and clinical characteristics, and phylogenetic analyses. Our work included a balanced study population with respect to gender, but was largely a young-aged population which is expected for a university. Also, the proportion of asymptomatic and symptomatic individuals was roughly the same for both Delta and Omicron variants. A majority of the 793 individuals (722 or 91.0 %) were considered vaccinated per our definition. There were no significant differences across vaccinated individuals with respect to Delta and Omicron variants, although a much higher percentage of individuals were boosted and had Omicron, but this is expected as booster vaccinations became widely available during this time period. Additionally, more individuals who had Omicron also had a previous infection more than 90 days prior to the latest positive diagnosis, although this is also expected as more time had elapsed, and thus there are more chances to become infected.

The Delta and Omicron variants appeared successively and rapidly as VOCs in the United States, and we observed this pattern of evolution of SARS-CoV-2 variants within our sample population from August 2021 to April 2022 (Fig. 1). Following the global emergence of Omicron variant in November 2021, the highly transmissible variant quickly replaced Delta within our Purdue study community. Specifically, for cases collected between the second week of the study and the second week of December 2021, Delta accounted for 100 % proportion of cases. The first Omicron variant case was detected through sequencing during the third week of December 2021, where 66.7 % of cases sequenced from that week were already classified as Omicron and the rest were Delta (Fig. 1). By the fourth week of December 2021, Delta samples only accounted for 19.0 % during this week and then dropped to undetectable levels in the third week of January 2022. These results are consistent with those observed in other university settings, as well as overall patterns recorded both nationally and across the globe [20, 36–38, 67, 68], although studies investigating this particular replacement are limited in number.

To further show the clear replacement of Delta variant by Omicron variant through time, we created a Bayesian phylogeny and present the time-informed Maximum Clade Credibility (MCC) tree (Fig. 2). Moreover, to shed light on the 793 SARS-CoV-2 samples from the university and contextualize them with nationwide and international samples, we conducted phylogenetic analyses and utilized a parsimony score-based method to investigate relationships between the populations of SARS-CoV-2 viruses at Purdue and viral populations outside this community.

When looking at the BaTS results from all countries for Delta variant, it was observed that the USA accounted for almost all of the total virus migration into the Purdue community, whereas for Omicron variant, the United States score represented slightly more than half of the total. In this latter case, there were 12 other countries with parsimony scores>1.0, with the United Kingdom, Poland, and India following the USA. In contrast, for Delta variant, there were only two other countries in addition to the USA with summed PS scores>1.0. When looking more closely at BaTS results from the United States, for Delta variant, we observed that the greatest support of transmission occurred in-state, with Indiana accounting for more than half of the score, followed by Illinois, California, and Ohio. In contrast, the BaTS results for Omicron variant showed that the highest value was from California, but this was was not much higher than Indiana, Illinois, and Tennessee. Our findings of 1) a wider range of potential geographic connections for Omicron viruses on our campus compared to the Delta variant and 2) the likelihood of a more significant impact from international and out-of-state migration, rather than within Indiana, in the emergence of Omicron variant at Purdue, have important implications from a public health perspective. These results highlight the benefits of real-time monitoring for variant detection, and provide institutional awareness of the effects of open campus travel policies, which subsequently could influence future mitigation and control strategies. Placing these results in context with the available limited travel history data collected from the patients (Fig. S2), for Delta variant, Illinois and Ohio were the most common travel reported. In the case of Omicron variant, Illinois was also the most prevalent travel reported, followed by Florida and India. These results are generally in accordance with the findings from our BaTS analyses, especially for Delta and less so for Omicron, which is consistent with a more rapid geographic diffusion of Omicron; however, it is important to remember that this is based off the limited travel history with which we were provided.

While our study provides valuable insight into rapid transition from Delta to Omicron variants in a campus community, our results should be interpreted in view of several limitations. First, there was no routine active surveillance carried out for vaccinated individuals to capture the entire campus population transmission landscape, and there was not sufficient capacity to perform genomic sequencing of more cases. Additionally, we did not have complete metadata provided by all cases included in the study, and thus we relied on patient reporting of correct information, such as vaccination dates and travel history. We also acknowledge that the analyses performed here reflect a generally healthy population with a young median age, and thus generalisations to less healthy and older populations should not be made.

Furthermore, Delta variant was identified by sequencing as having emerged in December of 2020, and our study investigated introduction of this variant 8 months into its establishment in communities, so this could have partly impacted our findings with respect to introduction events; on the other hand, we explored Omicron variant just at its emergence. In our use of the PS-based association statistic, we tracked PS score differences that were greater than 0.05. This conservative cut-off likely accepts some connections that are poorly supported or non-existent. This is because phylogenetic analysis of datasets with low phylogenetic signal, such as those with many samples that have identical sequences spread across multiple geographic regions during the same period, will distribute probability across these regions relatively uniformly, giving them non-zero affiliations. This is clearly true of SARS-CoV-2, which has unprecedented levels of sampling through time and space. We therefore choose to limit our interpretation to the largest PS difference values, as they correspond to the most supported and most significant epidemiological connections between Purdue and other regions. Notably, this is supported by an analysis of Gamma variants from Purdue, in which large PS difference scores correlated with high Bayesian support for connection between geographic locations [19].

Importantly, the investigation of viral migration between geographic regions from phylogenies must be done cautiously. Due to incomplete sampling, it is very unlikely that complete transmission chains can be established, and thus intermediate transmission events connecting two closely related viruses sampled from different geographic regions are unknowable. Furthermore, the incompleteness of sampling may in many cases preclude the establishment of the polarity of migration. Whilst our results demonstrate the existence of transmission networks between closely related viruses from different geographic regions, we caution that they should not be interpreted as direct measures of migration, but rather as indicative of the relative contribution of long-distance transmission to the local spread of viruses at Purdue.

Finally, we were concerned that our curation of the Omicron datasets for the cluster analyses, in which we eliminated samples that were identical by geographic state, sequence, and date to reduce redundancy and computational requirements, may have introduced a bias against Indiana and for increased geographic dispersion in our parsimony analysis of viral migration. However, when comparing the geographic locations of datasets before and after curation, we found that samples from Indiana were a larger proportion of all samples after removing duplicates for most cluster datasets (see Table S9). This pattern suggests that the differences in geographic connectedness we inferred for Delta and Omicron sequences from Purdue was not an artefact introduced by data curation, and our estimates of geographic connectedness accurately reflect patterns of SARS-CoV-2 circulation into Purdue University.

## Conclusion

Genomic surveillance of SARS-CoV-2 at Purdue University captured the rapid transition from Delta variant to Omicron variant across the West Lafayette campus. This mirrors the emergence of Omicron and its replacement of Delta observed globally, though this study is the first to document the rapidity with which a previously established variant is wholly displaced in the context of a university community emerging from COVID-19 mitigation efforts and with high vaccination levels. We identified a more diverse set of possible geographic origins for Omicron viruses on campus relative to Delta, and a potentially larger role for international and out-of-state (i.e. not Indiana) migration in the establishment of Omicron variants at Purdue. Notably, this pattern is consistent with the observed rapid diffusion of Omicron through populations as it displaced existing variants. Importantly, this study highlights the effects of diffuse networks connecting universities to geographically diverse locations on the circulation of highly transmissible respiratory viruses and the spread of new variants. Future analyses may profitably build on this work by studying the temporal dynamics of international contributions to local epidemics, the role highly connected universities in the transmission of viruses between less-cosmopolitan populations, and how collection date and incubation time may inform travel and isolation/recovery policies for universities or other similarly large and connected organisations.

## Supplementary Data

Supplementary material 1

Supplementary material 2
