# Supplementary material for: Investigating the rise of Omicron variant through genomic surveillance of SARS-CoV-2 infections in a highly vaccinated university population
Source: Microb Genom. 2024 Feb 9;10(2):001194. doi: 10.1099/mgen.0.001194 (PMC10926704; doi:10.1099/mgen.0.001194)
Supplement: Supplementary material 2 [file mgen-10-1194-s001.pdf]

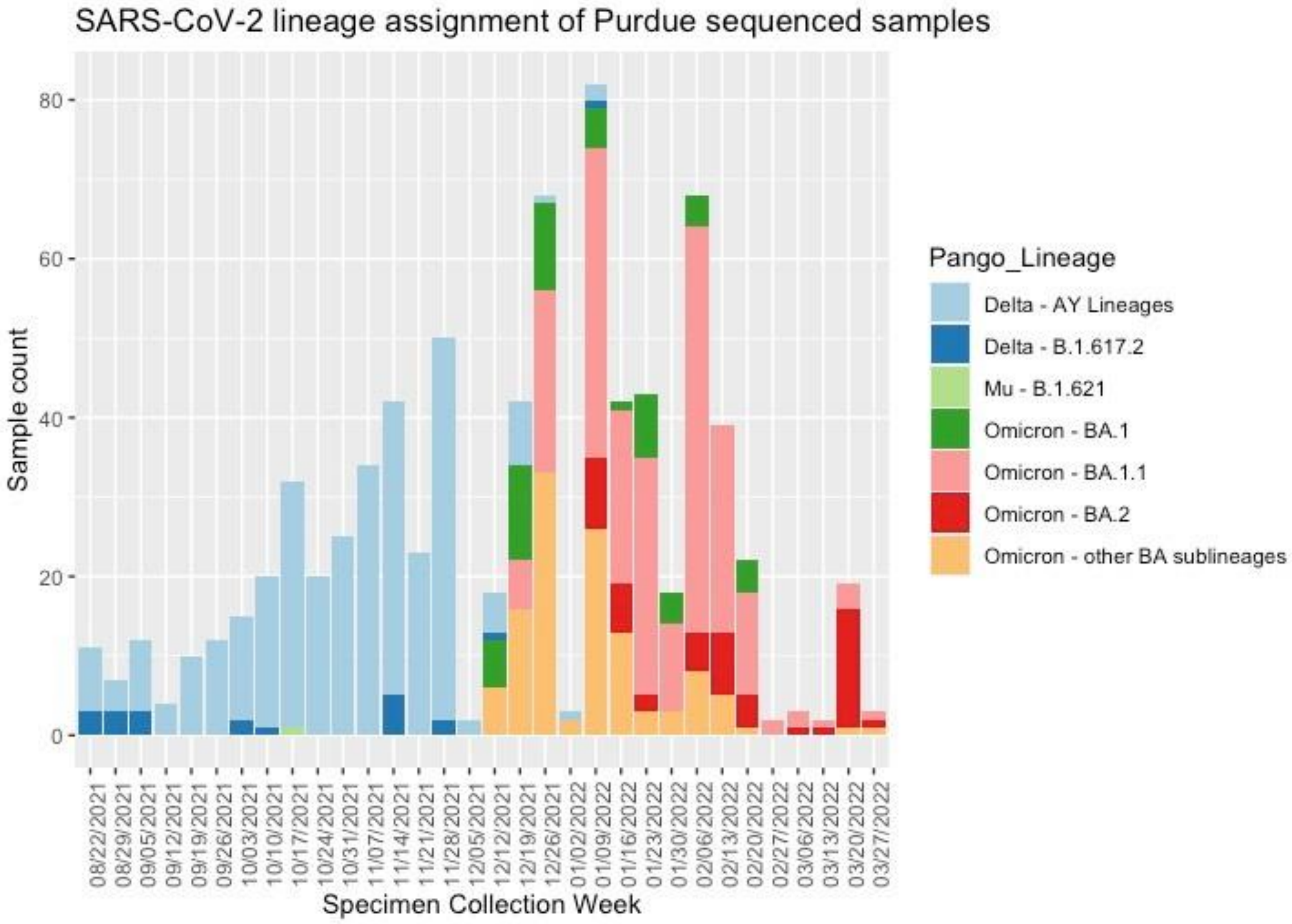

Supplementary Figure 1. Weekly counts of positive sequenced cases and their identified lineage assignment during the 32-week study period (August 22, 2021 to April 2, 2022) on Purdue's campus.

Each colour and shade represent a different PANGO lineage, with emphasis on the WHO variants identified as Variants of Concern (VOCs), Variants Being Monitored (VBMs), etc. The category labelled as "Omicron – other BA sublineages" represents a combination of sublineages of BA.1, BA.1.1, and BA.2 as they were identified through Pango_Lineage (Pango v.4.0.1 PLearn-v1.2.133) at the time of the study (April 2022). Important weeks on the Purdue University calendar are indicated (coinciding with academic campus breaks, etc.)

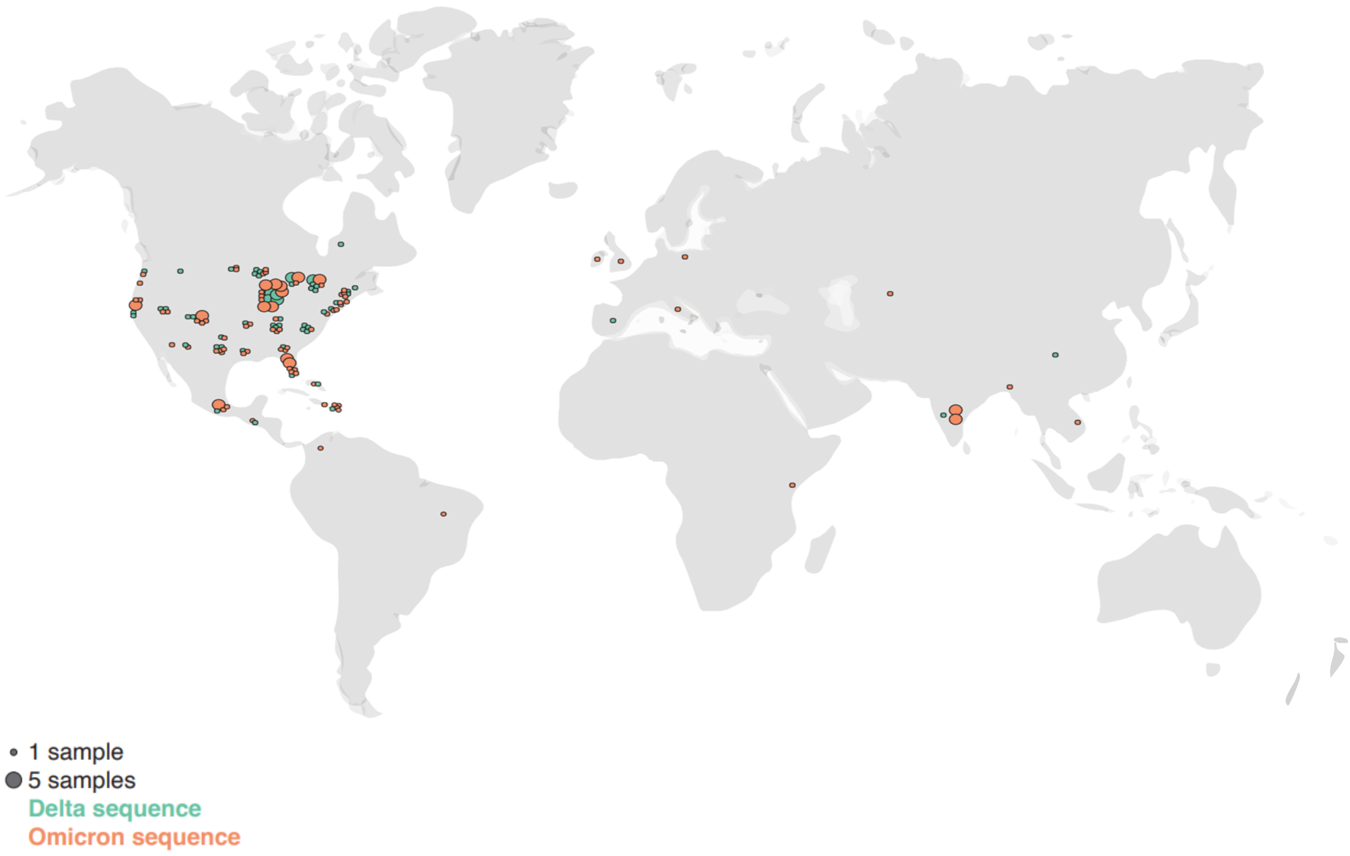

Supplementary Figure 2. World map of reported data of travel history from collected and sequenced samples in the Purdue Community.
The green dots denote samples confirmed to be Delta sequences, and the orange dots were confirmed Omicron sequences. Larger dots represent 5 samples, while smaller dots are 1 sample, and all samples are depicted in the map with approximate locations from self-reported data.
